# Understanding the Foreign Body Response via Single-Cell Meta-Analysis

**DOI:** 10.3390/biology13070540

**Published:** 2024-07-18

**Authors:** Norah E. Liang, Jennifer B. Parker, John M. Lu, Michael Januszyk, Derrick C. Wan, Michelle Griffin, Michael T. Longaker

**Affiliations:** 1 Hagey Laboratory of Pediatric Regenerative Medicine, Stanford University School of Medicine, Stanford, CA 94305, USA; nliang@stanford.edu (N.E.L.); jparker6@stanford.edu (J.B.P.); johnlu@stanford.edu (J.M.L.); januszyk@stanford.edu (M.J.); dwan@stanford.edu (D.C.W.); mgriff12@stanford.edu (M.G.); 2 Division of General Surgery, Department of Surgery, Stanford University School of Medicine, Stanford, CA 94305, USA; 3 Institute for Stem Cell Biology and Regenerative Medicine, Stanford University School of Medicine, Stanford, CA 94305, USA; 4 Division of Plastic and Reconstructive Surgery, Department of Surgery, Stanford University School of Medicine, Stanford, CA 94305, USA

**Keywords:** foreign body response, fibroblasts, macrophages, scRNA-seq, meta-analysis

## Abstract

**Simple Summary:**

When a medical implant or electrode is placed, the body often evokes an immune response in an attempt to remove it. As it fails to do so, this inflammatory response leads to a chronic fibrosis, wherein the material is walled off from the body with scar tissue. This phenomenon is known as foreign body response (FBR) and poses a significant burden, as it often impacts the implant’s function and longevity. Single-cell RNA sequencing (scRNA-seq), a technique evaluating the genes transcribed at a single-cell level within a given sample, has been used to identify targets to reduce the extent of fibrosis caused by FBR. We performed a meta-analysis collecting all available FBR mouse scRNA-seq studies to identify gene signatures specific to FBR across different models and anatomic locations. Through our integrated analysis, we identified specific groups of fibroblasts and macrophages associated with FBR, characterizing their associated genes and signaling pathways. Fibroblasts enriched in FBR had increased pro-fibrotic signatures, while macrophages enriched in FBR had both increased pro-fibrotic and pro-inflammatory profiles. Collectively, our meta-analysis identifies common cell-specific gene signatures in FBR and provides potential therapeutic targets for patients requiring long-term implanted biomedical devices.

**Abstract:**

Foreign body response (FBR) is a universal reaction to implanted biomaterial that can affect the function and longevity of the implant. A few studies have attempted to identify targets for treating FBR through the use of single-cell RNA sequencing (scRNA-seq), though the generalizability of these findings from an individual study may be limited. In our study, we perform a meta-analysis of scRNA-seq data from all available FBR mouse studies and integrate these data to identify gene signatures specific to FBR across different models and anatomic locations. We identify subclusters of fibroblasts and macrophages that emerge in response to foreign bodies and characterize their signaling pathways, gene ontology terms, and downstream mediators. The fibroblast subpopulations enriched in the setting of FBR demonstrated significant signaling interactions in the transforming growth factor-beta (TGF-β) signaling pathway, with known pro-fibrotic mediators identified as top expressed genes in these FBR-derived fibroblasts. In contrast, FBR-enriched macrophage subclusters highly expressed pro-fibrotic and pro-inflammatory mediators downstream of tumor necrosis factor (TNF) signaling. Cell–cell interactions were additionally interrogated using *CellChat*, with identification of key signaling interactions enriched between fibroblasts and macrophages in FBR. By combining multiple FBR datasets, our meta-analysis study identifies common cell-specific gene signatures enriched in foreign body reactions, providing potential therapeutic targets for patients requiring medical implants across a myriad of devices and indications.

## 1. Introduction

Foreign body response (FBR) poses a significant clinical problem for the growing population of patients reliant upon implantable medical devices and technologies [1]. FBR is characterized by an immunologic reaction to implanted material and fibrous encapsulation of the implant. This reaction is ubiquitous across a wide range of biomaterials and anatomic locations [1,2,3]. While previous studies have implicated subpopulations of macrophages and fibroblasts, as well as profibrotic pathways including transforming growth factor beta (TGF-β) and yes-associated protein 1 (YAP1), in driving FBR pathogenesis, these findings are limited by individual single-cell RNA sequencing (scRNA-seq) experiments from a single FBR model with limited biological replicates [4,5,6,7,8,9].

At present, few studies have leveraged scRNA-seq technology to characterize the subpopulations of cells involved in FBR [6,7,8,9]. The current limited understanding of FBR is based upon available genomic data derived from a handful of FBR mouse models. To our knowledge, there has yet to be a scRNA-seq dataset published using human-derived FBR cells. In the following study, we combine all publicly available FBR scRNA-seq datasets between January 2019 and January 2023 to further evaluate the dominant cell populations and pathways involved in FBR. By collating these data, we seek to provide insights into potential targets for treating FBR across all anatomic locations, providing targets for future studies that have the potential to revolutionize healthcare technology.

## 2. Materials and Methods

### 2.1. Screening of FBR-Related scRNA-seq Datasets

All publicly available FBR-related scRNA-seq datasets were identified by searching the Gene Expression Omnibus (GEO) and PubMed databases. The following search terms were used:

**GEO**: (“implant” OR “Foreign Body*” OR “FBR”) AND ((“single cell*” OR “single-cell*” OR “scRNA*” OR “sc-RNA*”) OR (“single nuc*” OR “single-nuc*” OR “snRNA*” OR “sn-RNA*”))
**PubMed**: (“implant” OR “Foreign Body*” OR “FBR”) AND ((“single cell*” OR “single-cell*” OR “scRNA*” OR “sc-RNA*”) OR (“single nuc*” OR “single-nuc*” OR “snRNA*” OR “sn-RNA*”))

The datasets identified were then manually screened for specific inclusion criteria. We included only datasets derived from droplet-based scRNA-seq platforms (10X Genomics, Drop-Seq, etc.) and those that included cells harvested from FBR models (could be from any anatomic location). Details on species, FBR model, mouse genotype, time point, anatomic region, and pre-sequencing cell processing were collected (Table 1). The mouse datasets were then further screened to exclude 1 redundant dataset, 1 sample without cells derived from an FBR capsule, and 4 samples where the mouse that was implanted with a foreign body also underwent concurrent anti-fibrotic or pro-fibrotic treatment [6,7,8,10].

### 2.2. Integration of FBR Datasets 

The count data for all cells from all scRNA-seq samples of interest were merged using Seurat version 5 (v5). The 4 layers in the combined object were then joined using the JoinLayers function, and the resulting object was integrated using the Harmony integration method [11]. The following steps were then performed using Seurat v5 functions with default parameters: NormalizeData, FindVariableFeatures, ScaleData, RunPCA, IntegrateLayers, RunUMAP, FindNeighbors, and FindClusters. The subsequent object was used for downstream analysis. 

## 3. Results

### 3.1. Identification of Major Cell Types Involved in FBR

**Figure 1:**
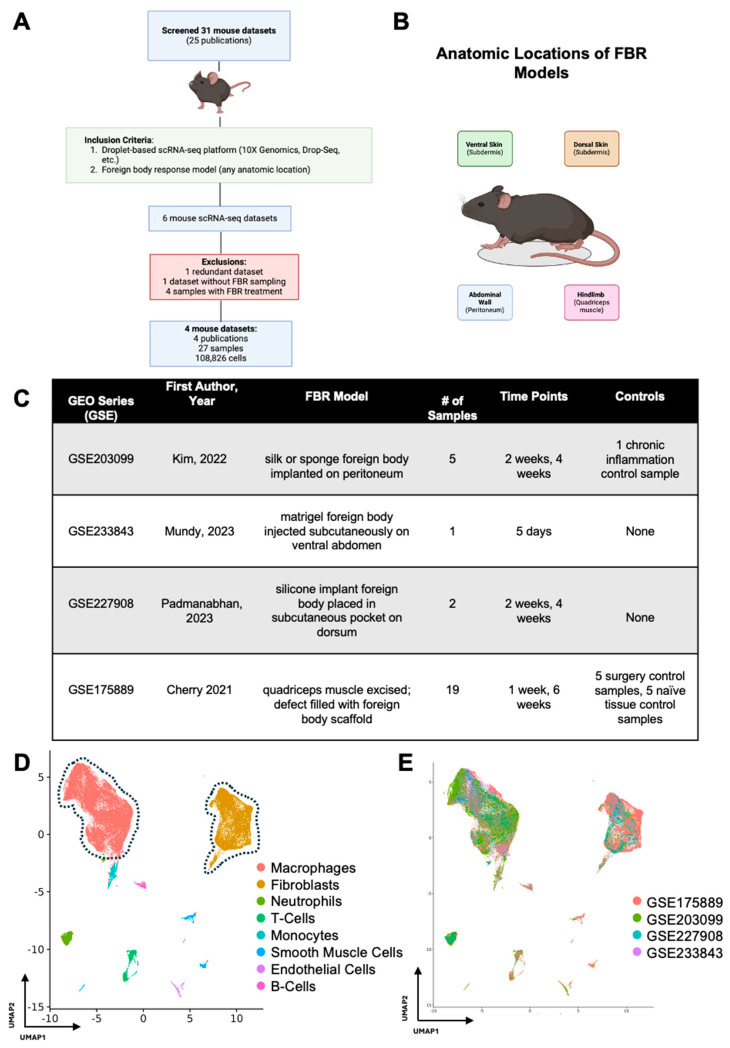
Meta-analysis workflow and data integration. (**A**) All publicly available foreign body response (FBR)-related single-cell RNA sequencing (scRNA-seq) datasets were identified by searching the Gene Expression Omnibus (GEO) and PubMed databases. The identified datasets were then manually screened for inclusion (green box) and exclusion (red box) criteria. (**B**) Schematic anatomic locations of the various FBR models included in the meta-analysis. (**C**) Experimental details associated with each GEO series [6,7,8,9]. (**D**) Uniform manifold approximation and projection (UMAP) of scRNA-seq data from all datasets included in the meta-analysis, colored according to cell type. (**E**) UMAP of scRNA-seq data from all datasets included in the meta-analysis, colored according to GEO accession number.

After initial screening, our search terms returned 31 FBR scRNA-seq datasets corresponding to 25 publications, all of which were derived from mouse cells (Figure 1A). A total of four mouse FBR datasets derived from four publications and 27 mouse samples met our inclusion criteria, comprising a total of 108,826 cells across four different anatomic locations and five unique time points (Figure 1B,C). [6,7,8,9] For their model, Kim et al. implanted a silk or sponge foreign body intra-abdominally onto the peritoneal wall and harvested FBR tissue at 2 and 4 weeks after surgery [6]. Mundy et al. injected a matrigel foreign body into the subdermis tissue of the ventral abdomen and analyzed the tissue on postoperative day (POD) 5 [7]. Padmanabhan and colleagues similarly examined FBR in the subdermis by placing a silicone implant into a subcutaneous pocket on the mouse dorsum [8]. Finally, Cherry et al. utilized an FBR model whereby the quadriceps muscle on the murine hindlimb was excised, the defect was filled with either a biologic or synthetic foreign body scaffold, and tissue was harvested at 1 and 6 weeks [9].

In our study, there were 11 samples derived from two studies which were identified as controls within those respective studies. Kim et al. performed a thioglycollate lavage of the peritoneum and collected elicited macrophages as a “chronic inflammation control”. As this sample did not include peritoneal cells (where the foreign body was implanted), it was omitted from further analysis [6]. Cherry et al. included two FBR control conditions in their study with five samples each: (1) the first consisted of naïve, excised quadriceps muscle (designated “Tissue Control”), and (2) the second was excised scar tissue following quadriceps muscle removal and saline injection into the defect area rather than a foreign body scaffold (designated “Surgery Control”) [9]. The two remaining studies did not include FBR controls [7,8].

Following integration of our 27 mouse samples, we identified two predominant cell populations involved in FBR (Figure 1D). As expected, macrophages and fibroblasts comprised the majority of cell types across all studies (identified by GEO series number (GSE)) included in our meta-analysis, irrespective of the FBR model and its anatomic location (Figure 1E). 

### 3.2. Detailing the Fibroblast Subpopulations Driving FBR

**Figure 2:**
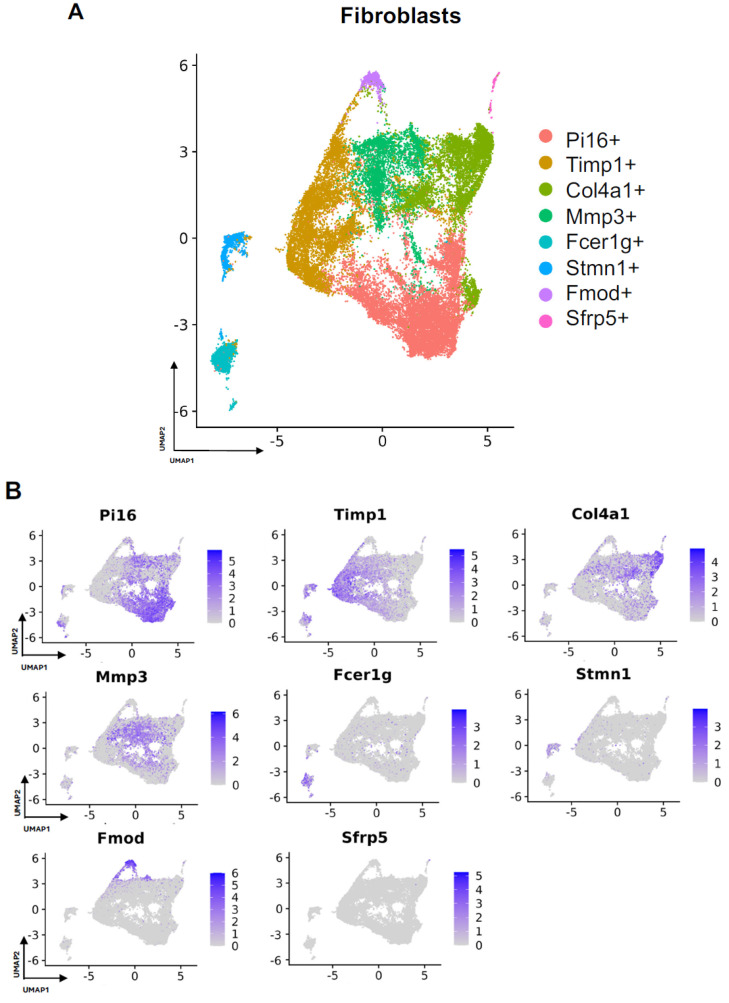
Defining fibroblast subpopulations in FBR. (**A**) Uniform manifold approximation and projection (UMAP) of fibroblasts annotated in silico by differential expression of subcluster-defining genes (Pi16+, Timp1+, Col4a1+, Mmp3+, Fcer1g+, Stmn1+, Fmod+, Sfrp5+). (**B**) UMAP of fibroblasts colored according to expression levels of subpopulation-defining genes.

**Figure 3:**
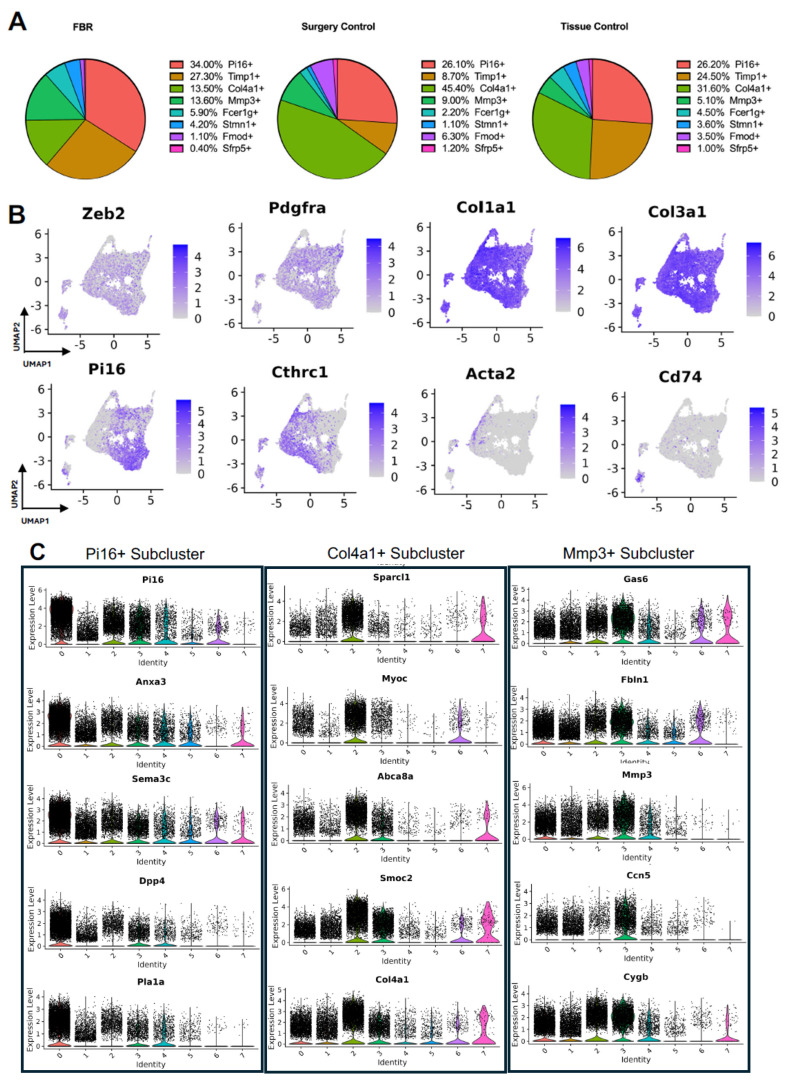
Details of fibroblast subpopulations driving FBR. (**A**) Proportional distribution of fibroblast subclusters from FBR samples (left), surgery control samples (center), and tissue control samples (right). (**B**) Uniform manifold approximation and projection (UMAP) of fibroblasts colored by expression level for canonical fibroblast markers. (**C**) Violin plots displaying expression levels of the top five genes within each of the Pi16+, Col4a1+, and Mmp3+ fibroblast subclusters. Each violin plot additionally illustrates the expression level of each gene across all other fibroblast subpopulations (0 = PI16+, 1 = Timp1+, 2 = Col4a1+, 3 = Mmp3+, 4 = Fcer1g+, 5 = Stmn1+, 6 = Fmod+, 7 = Sfrp+).

Subcluster analysis of all fibroblasts in our combined dataset revealed eight distinct subpopulations (Figure 2A), with representation of all subclusters across all four studies (Supplemental Figure S1A). For all fibroblast subpopulations, we identified the top differentially expressed gene in each subpopulation (expressed by at least 70% of the subcluster and less than 40% of the other subclusters). Subcluster-specific gene expression is shown in Figure 2B, denoted by Timp1+, Col4a1+, Mmp3+, Fcer1g+, Stmn1+, Fmod+, and Sfrp5+. To interrogate which fibroblast subpopulations may drive FBR, we compared the proportions of each subcluster in FBR cells to those derived from our “Surgery Control” and “Tissue Control” conditions (Figure 3A). Overall, there was a greater proportion of Pi16+ and Mmp3+ fibroblasts in the “FBR” condition compared to the two control conditions. Conversely, there was a relatively decreased proportion of Col4a1+ cells in the FBR condition compared to the two control conditions. Overall, all fibroblast subclusters expressed canonical fibroblast markers such as zinc finger E-box binding homeobox 2 (Zeb2), platelet-derived growth factor receptor alpha (Pdgfra), collagen type I alpha 1 chain (Col1a1), and collagen type III alpha 1 chain (Col3a1) (Figure 3B). Some subclusters expressed relatively more peptidase inhibitor 16 (Pi16), collagen triple helix repeating containing 1 (Cthrc1), actin alpha 2 (Acta2), and cluster of differentiation 74 (Cd74) than others, with these markers indicating different subtypes of fibroblasts.

To further classify the fibroblast subpopulations involved in FBR, we examined differential gene expression by subcluster. Subcluster pathway and gene ontology (GO) analyses using EnrichR further elucidated fibroblast identity and functionality [12,13]. We focused our analysis specifically on subclusters that were enriched (Pi16+ and Mmp3+ subclusters) or depleted (Col4a1+ subcluster) in the FBR condition. Pathways and GO terms associated with each relevant cluster are illustrated in Supplemental Figure S1B, with the top five genes from each cluster plotted in Figure 3C. The Pi16+ subcluster was enriched for “complement and coagulation cascade” pathways and GO terms associated with “TGF-β activity”, “growth factor binding”, “actin cytoskeleton”, and “collagen-containing extracellular matrix (ECM).” The most highly expressed genes in the Pi16+ subcluster included peptidase inhibitor 16 (Pi16), annexin A3 (Anxa3), semaphorin 3c (Sema3c), dipeptidyl peptidase (Dpp4), and phospholipase A1 (Pla1a). The Mmp3+ subcluster was associated with similar pathways to the Pi16+ subcluster, as well as those involving “type I collagen synthesis”, “nuclear factor kappa beta (NF-ΚB) signaling”, and pro-inflammatory “interleukin 6 (IL-6)”, as well as GO terms associated with “lysosomal lumen”, “regulation of smooth muscle cell proliferation”, and “external encapsulating structure organization”. Top genes from this cluster included growth arrest-specific gene 6 (Gas6), fibulin 1 (Fbln1), matrix metallopeptidase 3 (Mmp3), cellular communication network factor 5 (Ccn5), and cytoglobin (Cygb). In contrast, the Col4a1+ subcluster was relatively decreased in the FBR condition and was associated with “regulation of SMAD 2/3 signaling”, “mitogen-activated protein kinases (MAPK) signaling”, “osteopontin-mediated events”, and “adipogenesis” pathways, as well as “endoplasmic reticulum lumen”, “regulation of cell migration”, and “phosphatidylinositol 3-Kinase (PI3K) activity” GO terms. The Col4a1+ subcluster predominantly expressed the following genes: secreted protein, acidic and rich in cysteine (SPARC)-like 1 (Sparcl1), myocilin (Myoc), adenosine triphosphate (ATP)-binding cassette subfamily A member 8 (Abca8a), SPARC-related modular calcium-binding 2 (Smoc2), and collagen type IV alpha 1 chain (Col4a1). Additionally, among the enriched pathways in different subclusters, the Pi16+ and Mmp3+ subclusters shared common pathways involving “senescence and autophagy”, “complement and coagulation cascades”, “ECM organization”, and “collagen binding” (Table 2). In summary, we identified fibroblast subpopulations and their associated signaling pathways and downstream mediators that were increased in the setting of FBR.

### 3.3. Characterizing the Key Macrophage Subpopulations in FBR

**Figure 4:**
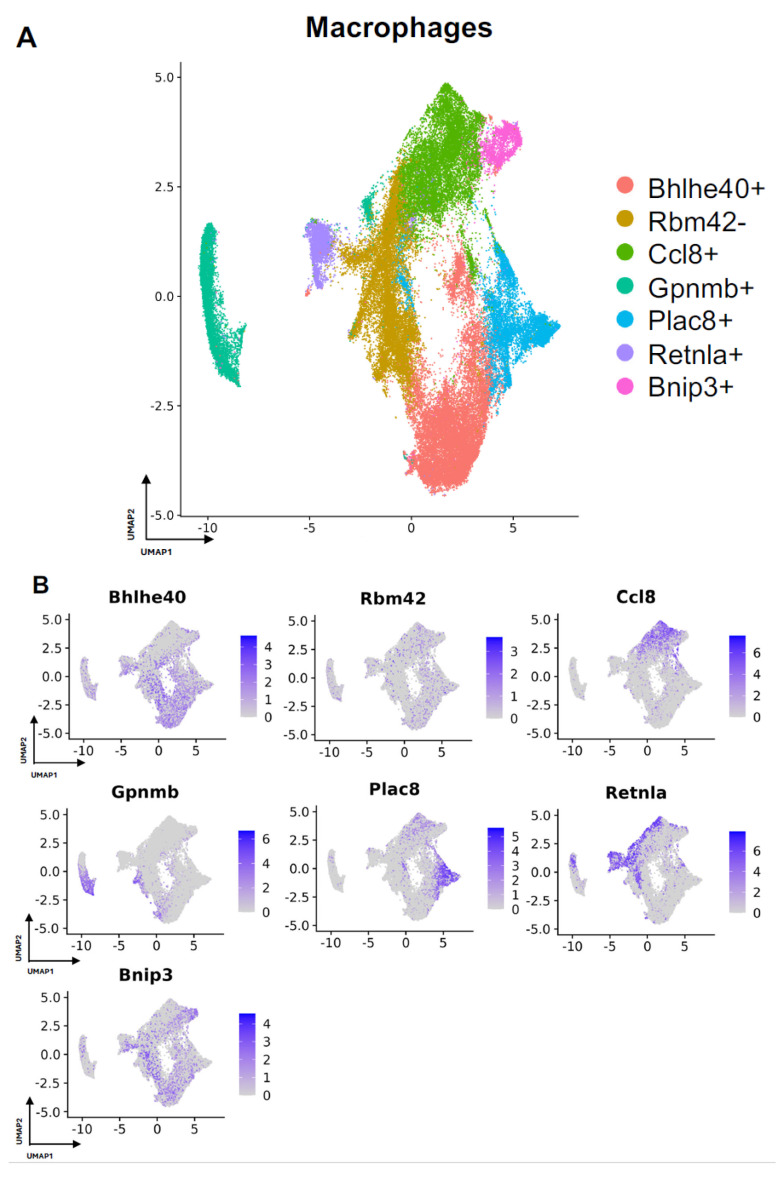
Defining macrophage subpopulations in FBR. (**A**) Uniform manifold approximation and projection (UMAP) of macrophages annotated in silico by differential expression of subcluster-defining genes (Bhlhe40+, Rbm42-, Ccl8+, Gpnmb+, Plac8+, Retnla+, Bnip3+). (**B**) UMAP of macrophages colored by expression level of subpopulation-defining genes.

Next, we turned our attention to characterization of the macrophage subpopulations in our meta-analysis. We identified 7 distinct subpopulations across the 4 studies (Figure 4A and Figure S2A). For all macrophage subpopulations, we identified the top differentially expressed gene in each subpopulation (expressed by at least 70% of the subcluster and less than 40% of the other subclusters), except for subcluster 1, which we identified with a gene that was expressed in less than 5% of subcluster 1 and more than 20% of other subclusters. Subcluster-specific gene expression is shown in Figure 4B, denoted by Bhlhe40+, Rbm42-, Ccl8+, Gpnmb+, Plac8+, Retnla+, and Bnip3+. The Bhlhe40+ and Bnip3+ subpopulations of macrophages were enriched in the setting of FBR.

**Figure 5:**
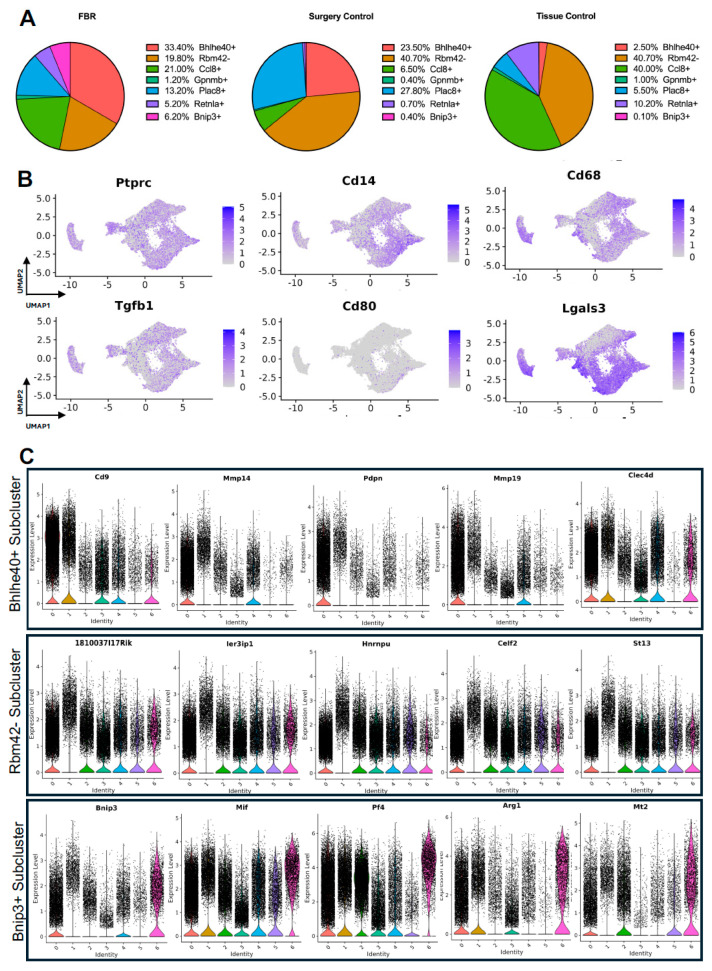
Detailing macrophage subpopulations driving FBR. (**A**) Proportional distribution of macrophage subclusters from FBR samples (left), surgery control samples (center), and tissue control samples (right). (**B**) Uniform manifold approximation and projection (UMAP) of macrophages colored by expression level for canonical macrophage markers. (**C**) Violin plots displaying expression levels of the top five genes within each of the Bhlhe40+, Rbm42-, and Bnip3+ macrophage subclusters. Each violin plot additionally illustrates the expression level of each gene across all other macrophage subpopulations. (0 = Bhlhe40+, 1 = Rbm42-, 2 = Ccl8+, 3 = Gpmb+, 4 = Plac8+, 5 = Retnla+, 6 = Bnip3+).

The comparison of FBR macrophage subpopulation proportions to those derived from control conditions was most notable for the relative enrichment of the Bhlhe40+ and Bnip3+ subpopulations and the relative depletion of the Rbm42- subcluster (Figure 5A), again with “Surgery Control” representing postoperative healing and “Tissue Control” representing naïve tissue. Common myeloid lineage/macrophage markers, including protein tyrosine phosphatase receptor c (Ptprc), cluster of differentiation 14 (Cd14), and cluster of differentiation 68 (Cd68), were expressed by the majority of macrophage subclusters (Figure 5B). Pro-inflammatory and macrophage activation genes, including Tgfb1, cluster of differentiation 80 (Cd80), and galectin-3 (Lgals3), were more selectively expressed in certain macrophage subpopulations.

As above, differential gene expression was analyzed to further distinguish macrophage subclusters of interest. Pathways and GO terms for the Bhlhe40+, Rbm42−, and Bnip3+ subclusters are illustrated in Supplemental Figure S2B, with the top genes from each subcluster plotted in Figure 5C. Analysis of the FBR-enriched Bhlhe40+ macrophage subcluster revealed similar themes to those identified in FBR-enriched fibroblast subpopulations. This included pathways involved in “ECM-receptor interaction”, “tumor necrosis factor (TNF) signaling”, the “hypoxia inducible factor 1 subunit alpha (HIF1A) transcription factor network”, and “regulation of actin cytoskeleton”, as well as GO terms associated with “phagocytosis”, “inflammatory response”, “focal adhesion”, and “regulation of cell migration”. The most highly expressed genes in the Bhlhe40+ subcluster included cluster of differentiation 9 (Cd9), matrix metalloproteinase 4 (Mmp4), podoplanin (Pdpn), matrix metalloproteinase 19 (Mmp19), and c-type lectin domain family 4 member D (Clec4d). Similarly, the Bnip3+ subcluster, which was also enriched in the setting of FBR, was associated with the “HIF1 signaling” and “oxidative phosphorylation” pathways, as well as the “fibroblast growth factor binding”, “cell-substrate junction”, and “cellular response to hypoxia” GO terms. Relevant genes in this cluster included B-cell leukemia/lymphoma 2 protein (Bcl-2), interacting protein 3 (Bnip3), macrophage inhibitory factor (Mif), platelet factor 4 (Pf4), arginase 1 (Arg1), and metallothionein 2 (Mt2). The Rbm42− subcluster, a subpopulation of macrophages found to be depleted in the setting of FBR, was associated with remodeling terms such as “messenger ribonucleic acid (mRNA) processing”, “spliceosome”, “wound healing”, and the chromatin-remodeling “B-WICH complex”. Highly expressed genes in this cluster included the RIKEN cDNA 1810037I17 gene (1810037I17Rik), immediate early response 3 interacting protein 1 (IER3IP1), heterogeneous nuclear ribonucleoprotein U (Hnrnpu), CUGBP, Elav-like family member 2 (Celf2), and suppression of tumorigenicity 13 (St13). In addition, analysis of the enriched pathways in all other subclusters demonstrated predominant overlap of “senescence and autophagy” pathways and “focal adhesion” and “cadherin binding” terms with FBR-enriched Bhlhe40+ and Bnip3+ subclusters (Table 3). Similarly to our fibroblast-centered analyses, a meta-analysis of the available scRNA-seq datasets allowed for the identification of macrophage subclusters enriched in FBR and their associated upregulated downstream effectors. 

### 3.4. CellChat Analysis of FBR Subpopulations

**Figure 6:**
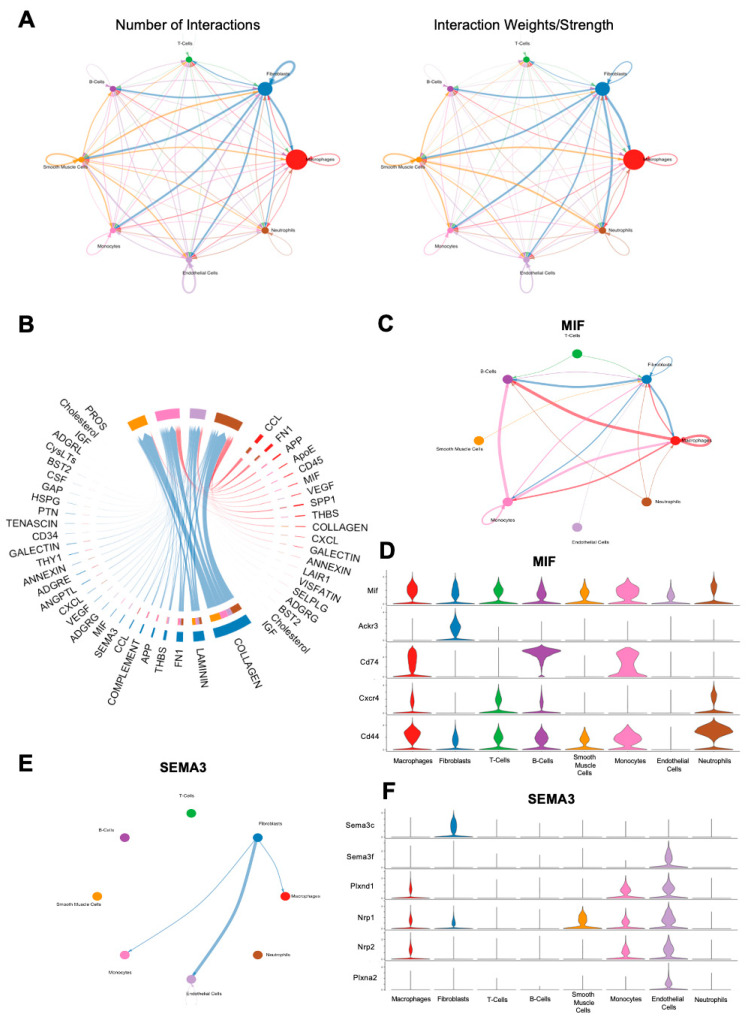
CellChat analysis of FBR cell subpopulations. (**A**) Number (**left**) and strength (**right**) of cell–cell interaction maps among cells from FBR capsules. Arrows depict cell populations from which signaling originates, with arrowheads depicting cell populations to which signaling interactions are directed. Thicker lines denote a greater number of interactions (**left**) and greater interaction weight (**right**). (**B**) Chord diagram displaying all significant interactions between macrophages and fibroblasts. Thicker lines denote stronger interactions. (**C**) Inferred macrophage migration inhibitory factor (MIF) signaling network in all cells. Thicker lines denote stronger interactions, with arrows illustrating the direction of signaling between cell types. (**D**) Expression distribution across cell types of MIF signaling-associated genes, illustrated on the y-axis (Mif, Ackr3, Cd74, Cxcr4, Cd44). Cell types are illustrated on the x-axis. Violin plots correspond to the expression level of each MIF-pathway mediator gene by each individual cell type. (**E**) Inferred Semaphorin-3 (SEMA3) signaling network in all cells. Thicker lines denote stronger interactions, with arrows illustrating the direction of signaling between cell types. (**F**) Expression distribution across cell types of SEMA3 signaling-associated genes, illustrated on the y-axis (Sema3c, Sema3f, Plxnd1, Nrp1, Nrp2, Plxna2). Cell types are illustrated on the x-axis. Violin plots correspond to the expression level of each SEMA3-pathway mediator gene by each individual cell type.

In order to examine cell–cell communication within the FBR microenvironment, we applied *CellChat*, a computational tool designed for this analysis, to all cells derived from the FBR condition [14]. In line with prior findings, *CellChat* suggested that the majority of cell–cell interactions centered around fibroblasts and macrophages (Figure 6A). Significant signaling pathways between these respective groups are shown in Figure 6B, with the most prominent interactions noted to be related to the collagen signaling pathways, as well as laminin and fibronectin. As Mif and Sema3c were noted as top expressed genes in the respective macrophage and fibroblast subclusters enriched in the setting of FBR, we investigated their associated signaling pathways using CellChat (Figure 3B and Figure 5B). Within the MIF signaling pathway, the predominant interactions were between fibroblasts, macrophages, B-cells, and monocytes (Figure 6C). Macrophages appeared to mediate MIF signaling via downstream mediators Cd44, C-x-c chemokine receptor type 4 (Cxcr4), Cd74, atypical chemokine receptor 3 (Ackr3), and Mif, whereas fibroblasts predominantly interacted with the MIF pathway via Mif, Ackr3, and Cd44 (Figure 6D). In contrast, CellChat analysis of the SEMA3 pathway showed primarily outgoing interactions from fibroblasts to macrophages, monocytes, and endothelial cells (Figure 6E). Of interest, fibroblast interactions in the SEMA3 pathway were mediated by Sema3c and neuropilin 2 (Nrp2), and macrophage interactions were mediated by neuropilin 1 (Nrp1) and plexin A2 (Plxna2), as well as Nrp2 (Figure 6F). Notably, neither the TNF nor the TGF-β pathways were found to show significant interactions upon CellChat analysis, despite being heavily implicated in our scRNA-seq analyses, as well as in other FBR studies [15,16,17,18,19].

## 4. Discussion

To our knowledge, this study represents the first meta-analysis of FBR-derived scRNA-seq data. We combined scRNA-seq samples from four different FBR models, each from a unique anatomical site. Fibroblasts and macrophages emerged as the predominant cell types in FBR, and our pooled data facilitated the identification of unique fibroblast and macrophage subpopulations universally enriched or depleted in the setting of implanted biomaterials. These analyses allowed us to examine FBR-associated gene expression on a more global level. By collating scRNA-seq data from all available FBR studies, we were able to identify common FBR gene signatures. In-depth pathway analysis revealed common themes of TNF signaling, ECM-associated terms, and inflammatory response.

In isolation, each study reported similar or related scRNA-seq findings on FBR using their respective models. Using a previously described mouse model of volumetric muscle loss, Cherry et al. constructed a single-cell atlas of the biomaterial microenvironment using both a biologic ECM scaffold and a synthetic polycaprolactone scaffold [9,20]. This was notable in that doing so allowed the authors to comment on differential gene expression with different biomaterials. They found that TNF signaling and inflammation were significantly increased in myeloid and endothelial cells in the setting of their synthetic FBR biomaterial compared to biologic-derived scaffold. The authors posited that this contributed to inhibition of wound healing in the setting of FBR. Additionally, endothelial cells derived from synthetic scaffold treatment were also associated with increased TGF-β signaling, and fibroblasts derived from synthetic treatment were associated with increased Myc signaling. These signaling pathways have previously been linked to fibrosis and chronic wounds, respectively [21,22]. Finally, they reported increased proportions of endothelial cells, mast cells, and anti-inflammatory macrophages with biological scaffold treatment, suggestive of increased vascularization and regeneration. Kim and colleagues focused on analyzing macrophage subsets derived from their peritoneal sponge and silk FBR models [6]. Similarly to Cherry et al., Kim and colleagues identified populations of myeloid-lineage cells which were increased in the setting of FBR, including: (1) a subpopulation of giant cells (GCs) enriched in the expression of pro-inflammatory genes and (2) another subpopulation of macrophage–epithelioid cells (EPCs) enriched in ECM organization terms. Mundy et al.’s investigation focused on investigating the effect of palovarotene on heterotopic ossification in a mouse model using Matrigel and recombinant human bone morphogenetic protein 2 (rhBMP-2) [7]. While FBR was not a focus of their study, we were able to include scRNA-seq data from their control sample to increase the generalizability of our meta-analysis findings. Finally, Padmanabhan and colleagues demonstrated that pathologic FBR was driven by Rac2 mechanotransduction signaling in myeloid cells [8]. These authors similarly noted activation of inflammatory markers in the setting of FBR, which was predominantly driven by myeloid cells and fibroblasts. 

Generally, our scRNA-seq meta-analysis revealed enrichment of pathways and GO terms associated with inflammation, collagen synthesis, ECM formation, and encapsulation in FBR-associated fibroblasts. In particular, our pooled data strongly implicated the TGF-β signaling pathway, with *Dpp4* and *Fbln1* found to be among the top expressed genes in FBR-derived fibroblasts. Both *Dpp4* and *Fbln1* are activated by TGF-β and mediate downstream pro-fibrotic activity [23,24]. Fibrosis and FBR are inextricably linked, with FBR felt to represent chronic fibrosis [1,25]. *Dpp4* is known play a role in fibrosis, having been described as a marker for pro-fibrotic dermal fibroblasts both in the setting of dermal scarring and in patients with systemic sclerosis [26,27,28]. Similarly, *Fbln1* encodes an ECM glycoprotein which is upregulated in the setting of pathogenic fibrosis, such as in diseases like idiopathic pulmonary fibrosis [29]. The role of TGF-β in fibrosis and FBR pathogenesis has previously been described, with prior studies noting enhanced transformation of fibroblasts to myofibroblasts, increased ECM formation, and increased fibroblast activation in the setting of TGF-β activation [17,30,31]. Our meta-analysis additionally implicated proinflammatory signaling pathways involving NF-ΚB and IL-6 in fibroblast-related FBR pathogenesis, with increased expression of associated genes *Anxa3* and *Gas6*. *Anxa3* is known to promote immune infiltration through the NF-ΚB signaling pathway and has previously been implicated as a key candidate gene in the progression of idiopathic pulmonary fibrosis [32,33]. Similarly, *Gas6* has been shown to become activated in the setting of inflammation across multiple tissue types and is associated with regulation of IL-6 signaling, ECM deposition, and fibrosis remodeling [34,35,36]. Altogether, these combined data point towards potential targets for fibroblast-related FBR pathogenesis.

Likewise, common FBR-associated macrophage pathways and GO terms were centered around ECM terms, inflammatory signaling, and phagocytosis. The TNF pathway is well established in terms of promoting inflammation, with multiple studies linking it to fibrosis as well [37,38,39]. Related genes that were highly expressed in these macrophage subclusters included *Cd9*, *Pdpn*, *Clec4d*, and *Mmp19*, all of which have separately been linked to fibrosis contexts outside of FBR. Rabhi et al. found that a Cd9+ subpopulation of macrophages isolated from the fat pads of mice fed a high-fat diet concurrently expressed Tnf-α, were notably expanded in the fibrotic stroma of white adipose tissue, and were a significant source of pro-fibrotic and proinflammatory factors [40]. *Pdpn*, another downstream mediator of TNF-α, encodes a transmembrane glycoprotein that has been linked to cell motility, with Pdpn-expressing cells thought to play an important role in post-myocardial infarction cardiac fibrosis [41,42,43]. Finally, *Clec4d* and *Mmp19* have been linked to pulmonary fibrosis, with *Clec4d* found to be highly expressed in lung tissue obtained from a mouse model of pulmonary injury and *Mmp19* found to induce monocyte infiltration in human and mouse fibrotic pulmonary disease [44,45]. Based on our meta-analysis, another highly implicated signaling pathway in FBR is the HIF1A pathway, with related downstream genes including *Bnip3* and *Mif*, which have been described in the pathogenesis of fibrosis. In renal interstitial fibrosis, *Bnip3* has been posited to activate autophagy and renal fibrosis as an effector of the HIF pathway in a mouse model of unilateral ureteral obstruction [46]. Like *Bnip3*, *Mif* is a hypoxia-inducible, HIF-dependent gene [47]. In a bleomycin-induced rat model of pulmonary fibrosis, Luo and colleagues demonstrated knockdown of *Mif* expression to be associated with inhibition of lung inflammation and fibrosis [48]. Several of these genes have been proposed as potential targets for pathologic fibrosis in organ-specific contexts. Given our findings, they may be reasonable targets for FBR as well.

In addition to the pathways described above, *CellChat* analysis of our combined FBR dataset revealed additional signaling pathways of potential interest. Although the analysis of fibroblast–macrophage interactions showed collagen-associated signaling to be most significant, *CellChat* additionally implicated Laminin and Fibronectin-1 (FN1) signaling. Laminins are a family of high-molecular-weight glycoproteins that play a key role in mediating interactions between cells and ECM proteins [49]. In the setting of neural implants, laminin coating of neural electrodes has been proposed as a method for improving biocompatibility by mitigating glial encapsulation and subsequent FBR [50,51]. In a percutaneous implant model, laminin coating of the implant was shown to improve the adhesion strength of the implant to the surrounding tissue without compromising biocompatibility [52]. Interestingly, Cao et al. found that implantation of foreign material coated in laminin-rich Matrigel into the peritoneal cavity of rats induced the most aggressive FBR response compared to material coated with multiple layers of elastin, collagen I, collagen III, or chitosan [53]. With respect to fibrosis, serum levels of laminin have been described to correlate positively with degree of fibrosis in multiple fibrotic liver diseases [54,55,56]. Conversely, laminin deficiency has also been associated with muscle and lung fibrosis in mouse models of muscular dystrophy and pulmonary fibrosis [57,58]. Like laminins, fibronectins are also ECM proteins that regulate cellular migration and adhesion [59]. With respect to FBR, fibronectin is known to play a role in macrophage adhesion and foreign-body giant cell formation [60]. In a mouse model of FBR with subcutaneous disc placement, deletion of plasma fibronectin in conditional knock-out mice resulted in alteration of the chronic fibrotic response, with a significantly increased number of foreign body giant cells associated with the implant, as well as increased capsule thickness seen in mice depleted of fibronectin [61]. The authors posited that the unexpectedly thickened capsule implicated plasma fibronectin in capsule remodeling. Generally, increased fibrosis has been linked to increased levels of fibronectin [62,63]. This may be explained by the observation that certain variants of fibronectin have been shown to sequester TGF-β in the ECM, leading to increased myofibroblast activation and collagen deposition [64]. Though both the laminin and fibronectin signaling pathways are broad, FBR pathogenesis is intimately tied to their related interactions, and as such, further interrogation may provide potential therapeutic avenues. 

Our meta-analysis does have limitations. First, there exist few established models for FBR in the literature. Of these, there are fewer scRNA-seq-based investigations of FBR, and those that exist were not all conducted using droplet-based scRNA-seq techniques, resulting in the small number of studies included in our meta-analysis. Notably, all identified FBR studies in our meta-analysis were based on cells isolated from mouse models, further limiting our meta-analysis to only one species. Additionally, we only included studies found on GEO, which may have caused us to omit data stored in other databases, such as the Broad’s Single Cell Portal or the China National GenBank DataBase (CNGBdb). Of the scRNA-seq samples included in our study, there were differences in FBR time points between studies, and we excluded samples that underwent proposed treatment of FBR in order to gain better transcriptomic clarity into the FBR condition alone. Finally, only one study included scRNA-seq data for purported control conditions, making it difficult to compare FBR datasets to control conditions, as not only is there no perfect control for FBR generally, but the included control samples may also be poor comparators to the FBR conditions in other models.

## 5. Conclusions

In conclusion, our meta-analysis provides a summary of the publicly available mouse-derived FBR scRNA-seq studies. By combining multiple investigations, we provide a generalizable perspective on the genomic underpinnings of the FBR condition. In our study, we provide a detailed investigation into the signaling pathways and downstream mediators enriched in the fibroblast and macrophage subpopulations driving FBR. Our results provide an initial resource against which to interpret future transcriptomic studies of FBR and potential targets for downstream validation.

## Figures and Tables

**Table 1:** Characterization of single-cell RNA sequencing samples curated from the Gene Expression Omnibus (GEO).

GSE	GSM	Species	Genotype	FBR Model	Condition	Treatment?	Time Point	Anatomic Region	Sorted?
GSE203099	GSM6153772	mouse	C57BL/6	control	Chronic Inflammation Control	N	NA	peritoneum	unsorted
GSE203099	GSM6153773	mouse	C57BL/6	sponge	FBR	N	2 weeks	peritoneum	unsorted
GSE203099	GSM6153774	mouse	C57BL/6	sponge	FBR	N	4 weeks	peritoneum	unsorted
GSE203099	GSM6153775	mouse	C57BL/6	silk	FBR	N	2 weeks	peritoneum	unsorted
GSE203099	GSM6153776	mouse	C57BL/6	silk	FBR	N	4 weeks	peritoneum	unsorted
GSE233843	GSM7437854	mouse	CD-1	matrigel only	FBR	N	5 days	subdermis	unsorted
GSE227908	GSM7110608	mouse	C57BL/6	implant	FBR	N	2 weeks	subdermis	unsorted
GSE227908	GSM7110609	mouse	C57BL/6	implant	FBR	N	4 weeks	subdermis	unsorted
GSE175889	GSM5350798	mouse	C57BL/6	ECM implant	FBR	N	1 week	quadricep	CD45-enriched
GSE175889	GSM5350799	mouse	C57BL/6	PCL implant	FBR	N	1 week	quadricep	CD45-enriched
GSE175889	GSM5350800	mouse	C57BL/6	Saline	Surgery Control	N	1 week	quadricep	CD45-enriched
GSE175889	GSM5350801	mouse	C57BL/6	control (sham)	Tissue Control	N	1 week	quadricep	CD45-enriched
GSE175889	GSM5350802	mouse	C57BL/6	ECM implant	FBR	N	1 week	quadricep	CD45-enriched
GSE175889	GSM5350803	mouse	C57BL/6	PCL implant	FBR	N	1 week	quadricep	CD45-enriched
GSE175889	GSM5350804	mouse	C57BL/6	Saline	Surgery Control	N	1 week	quadricep	CD45-enriched
GSE175889	GSM5350805	mouse	C57BL/6	control (sham)	Tissue Control	N	1 week	quadricep	CD45-enriched
GSE175889	GSM5350806	mouse	C57BL/6	ECM implant	FBR	N	1 week	quadricep	Fibroblasts;(CD45-CD19-CD31-CD29+)
GSE175889	GSM5350807	mouse	C57BL/6	PCL implant	FBR	N	1 week	quadricep	Fibroblasts;(CD45-CD19-CD31-CD29+)
GSE175889	GSM5350808	mouse	C57BL/6	Saline	Surgery Control	N	1 week	quadricep	Fibroblasts;(CD45-CD19-CD31-CD29+)
GSE175889	GSM5350809	mouse	C57BL/6	control (sham)	Tissue Control	N	1 week	quadricep	Fibroblasts;(CD45-CD19-CD31-CD29+)
GSE175889	GSM5350810	mouse	C57BL/6	ECM implant	FBR	N	6 weeks	quadricep	Fibroblasts;(CD45-CD19-CD31-CD29+)
GSE175889	GSM5350811	mouse	C57BL/6	PCL implant	FBR	N	6 weeks	quadricep	Fibroblasts;(CD45-CD19-CD31-CD29+)
GSE175889	GSM5350812	mouse	C57BL/6	Saline	Surgery Control	N	6 weeks	quadricep	Fibroblasts;(CD45-CD19-CD31-CD29+)
GSE175889	GSM5350813	mouse	C57BL/6	control (sham)	Tissue Control	N	6 weeks	quadricep	Fibroblasts;(CD45-CD19-CD31-CD29+)
GSE175889	GSM5350814	mouse	C57BL/6	ECM implant	FBR	N	1 week	quadricep	Macrophages from previously published dataset: (CD45+F4/80hi+Ly6c+CD64+)
GSE175889	GSM5350815	mouse	C57BL/6	PCL implant	Surgery Control	N	1 week	quadricep	Macrophages from previously published dataset: (CD45+F4/80hi+Ly6c+CD64+)
GSE175889	GSM5350816	mouse	C57BL/6	Saline	Tissue Control	N	1 week	quadricep	Macrophages from previously published dataset: (CD45+F4/80hi+Ly6c+CD64+)

**Table 2:** Overlapping EnrichR pathways and gene ontology terms between Pi16+ and Mmp3+ fibroblast subclusters and all other fibroblast subclusters.

	Timp1+	Col4a1+	Fcer1g+	Stmn1+	Fmod+	Sfrp5+
Wiki	Senescence and autophagy	Senescence and autophagy	X	X	Senescence and autophagy	Senescence and autophagy
KEGG	Complement and coagulation cascades ECM receptor interaction Focal adhesion	ECM receptor interaction Focal adhesion	Complement and coagulation cascades	X	ECM receptor interaction	ECM receptor interaction Focal adhesion
NCI	Beta1 integrin cell surface interactions	Beta1 integrin cell surface interactions	X	X	Beta1 integrin cell surface interactions	Beta1 integrin cell surface interactions
Biological	Cellular protein metabolic process ECM organization	ECM organization	X	X	ECM organization	ECM organization
Cellular	Endoplasmic reticulum lumen Proteinaceous ECM	Endoplasmic reticulum lumen	X	X	Endoplasmic reticulum lumen Proteinaceous ECM Microfibril	Endoplasmic reticulum
Molecular	Collagen binding Heparin binding	Collagen binding Heparin binding	X	X	Collagen binding Heparin binding	X

ECM, extracellular matrix. “X” denotes an absence of overlapping pathways/terms.

**Table 3:** Overlapping EnrichR pathways and gene ontology terms between Bhlhe40+ and Bnip3+ macrophage subclusters and all other macrophage subclusters.

	Rbm42−	Ccl8+	Gpnmb+	Plac8+	Retnla+
Wiki	X	Senescence and autophagy	X	Senescence and autophagy	Senescence and autophagy
KEGG	Oxidative phosphorylation	Carbon fixation	X	X	X
NCI	X	HIF-1-alpha transcription factor network	X	X	X
Biological	X	X	X	X	X
Cellular	Focal adhesion	Focal adhesion	Focal adhesion	X	Focal adhesion
Molecular	Cadherin binding RNA binding Proton-transporting ATP synthase activity	Cadherin binding	Cadherin binding	X	RNA binding

RNA, ribonucleic acid; ATP, adenosine triphosphate; “X” denotes an absence of overlapping pathways/terms.

## Data Availability

All scRNA-seq data used in this study are available from the Gene Expression Omnibus (GEO) using the following GEO series: GSE203099, GSE233843, GSE227908, and GSE175889.

## Supplementary Materials

The following supporting information can be downloaded at: https://www.mdpi.com/article/10.3390/biology13070540/s1, Figure S1: EnrichR pathways and Gene Ontology Terms for Meta-analysis Fibroblast Subclusters; Figure S2: EnrichR pathways and Gene Ontology Terms for Meta-analysis Macrophage Subclusters.
