# Supplementary material for: Understanding the Foreign Body Response via Single-Cell Meta-Analysis
Source: Biology (Basel). 2024 Jul 18;13(7):540. doi: 10.3390/biology13070540 (PMC11273435; doi:10.3390/biology13070540)
Supplement: Supplementary file 1 [file biology-13-00540-s001.zip › Supplemental Figure Legends_MDPI.pdf]

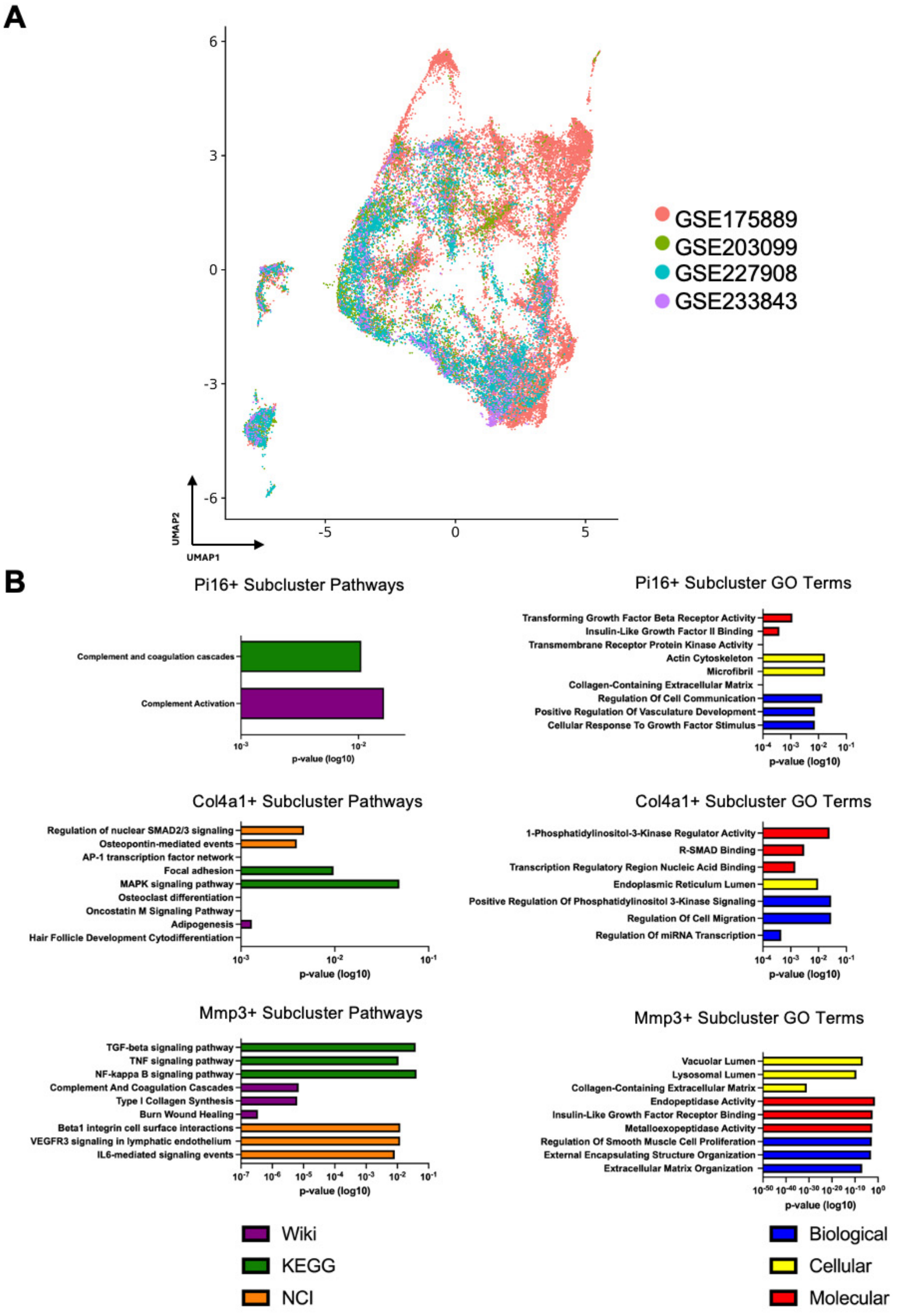

Figure S1: (**A**) Uniform-manifold approximation and projection (UMAP) of fibroblasts colored by Gene Expression Omnibus (GEO) accession number. (**B**) EnrichR analysis results illustrating Pathways (left) and Gene Ontology (GO) Terms (right) characteristic of cells in Pi16+ (top row), Col4a1+ (middle row), and Mmp3+ (bottom row) fibroblast

subclusters based on differential gene expression across fibroblasts. Pathways queried from Wiki (purple), KEGG (green), and NCI (orange). Gene ontology terms queried from Biological (blue), Cellular (yellow), and Molecular (red) processes. KEGG, Kyoto Encyclopedia of Genes and Genomes; NCI, National Cancer Institute.

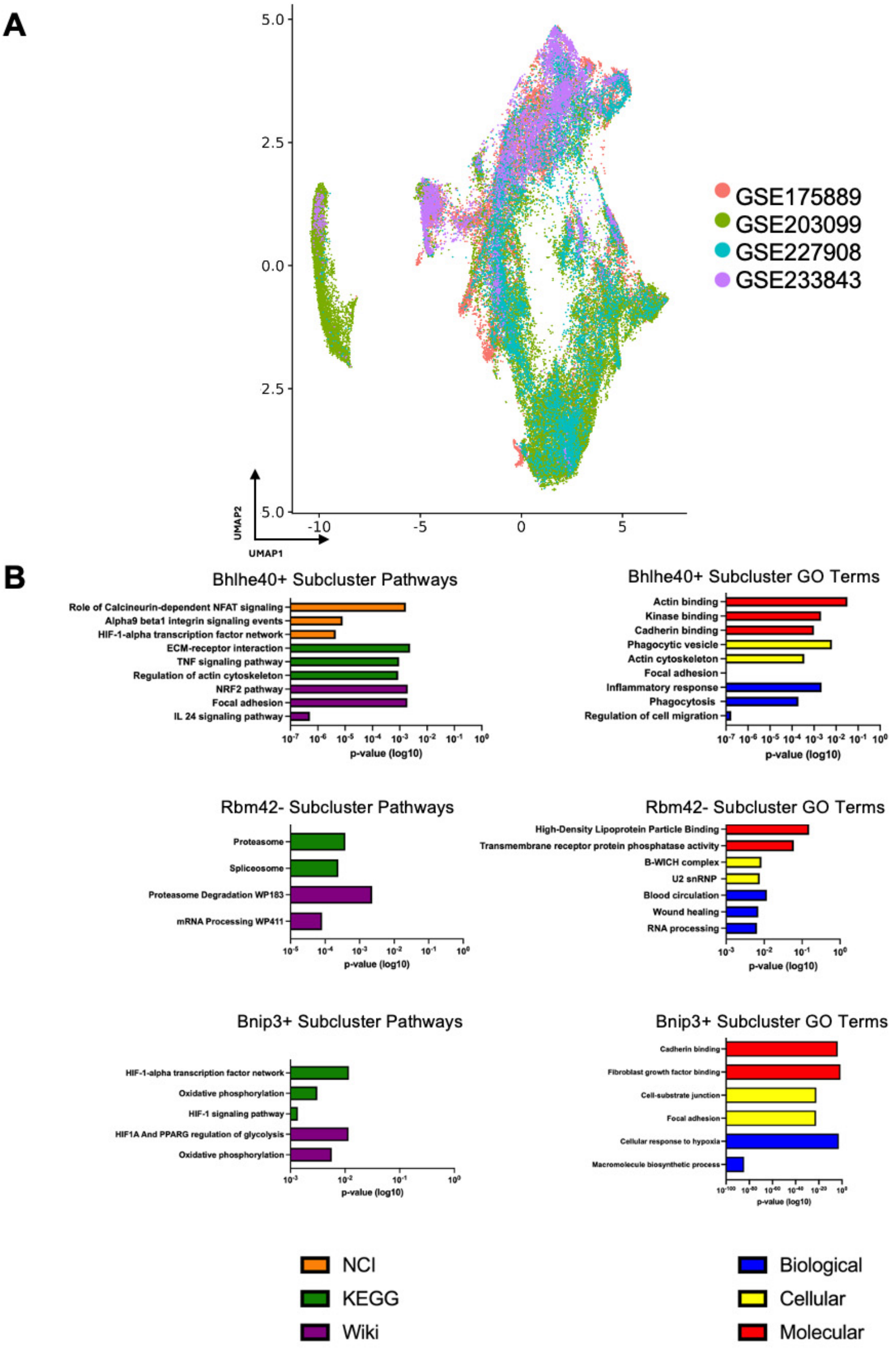

Figure S2: (**A**) Uniform-manifold approximation and projection (UMAP) of macrophages colored by Gene Expression Omnibus (GEO) accession number. (**B**) EnrichR analysis results illustrating Pathways (left) and Gene Ontology (GO) Terms (right) characteristic of cells in Bhlhe40+ (top row), Rbm42- (middle row), and Bnip3+ (bottom row) macrophage subclusters based on differential gene expression across macrophages. Pathways queried from Wiki (purple), KEGG (green), and NCI (orange). Gene ontology terms queried from Biological (blue), Cellular (yellow), and Molecular (red) processes. KEGG, Kyoto Encyclopedia of Genes and Genomes; NCI, National Cancer Institute.
